# Triboelectric and Piezoelectric Nanogenerators for Self-Powered Healthcare Monitoring Devices: Operating Principles, Challenges, and Perspectives

**DOI:** 10.3390/nano12244403

**Published:** 2022-12-09

**Authors:** Enrique Delgado-Alvarado, Jaime Martínez-Castillo, Luis Zamora-Peredo, Jose Amir Gonzalez-Calderon, Ricardo López-Esparza, Muhammad Waseem Ashraf, Shahzadi Tayyaba, Agustín L. Herrera-May

**Affiliations:** 1 Micro and Nanotechnology Research Center, Universidad Veracruzana, Boca del Río 94294, Veracruz, Mexico; 2 Cátedras CONACYT-Institute of Physic, Universidad Autónoma de San Luis Potosí, San Luis Potosí 78290, San Luis Potosí, Mexico; 3 Departamento de Física, Universidad de Sonora, Hermosillo 83000, Sonora, Mexico; 4 Department of Physics, Government College University Lahore, Lahore 54000, Pakistan; 5 Department of Computer Engineering, The University of Lahore, Lahore 54000, Pakistan; 6 Maestría en Ingeniería Aplicada, Facultad de Ingeniería de la Construcción y el Hábitat, Universidad Veracruzana, Boca del Río 94294, Veracruz, Mexico

**Keywords:** energy harvesting, green energy, internet of medical things, monitoring healthcare devices, piezoelectric nanogenerator, triboelectric nanogenerator

## Abstract

The internet of medical things (IoMT) is used for the acquisition, processing, transmission, and storage of medical data of patients. The medical information of each patient can be monitored by hospitals, family members, or medical centers, providing real-time data on the health condition of patients. However, the IoMT requires monitoring healthcare devices with features such as being lightweight, having a long lifetime, wearability, flexibility, safe behavior, and a stable electrical performance. For the continuous monitoring of the medical signals of patients, these devices need energy sources with a long lifetime and stable response. For this challenge, conventional batteries have disadvantages due to their limited-service time, considerable weight, and toxic materials. A replacement alternative to conventional batteries can be achieved for piezoelectric and triboelectric nanogenerators. These nanogenerators can convert green energy from various environmental sources (e.g., biomechanical energy, wind, and mechanical vibrations) into electrical energy. Generally, these nanogenerators have simple transduction mechanisms, uncomplicated manufacturing processes, are lightweight, have a long lifetime, and provide high output electrical performance. Thus, the piezoelectric and triboelectric nanogenerators could power future medical devices that monitor and process vital signs of patients. Herein, we review the working principle, materials, fabrication processes, and signal processing components of piezoelectric and triboelectric nanogenerators with potential medical applications. In addition, we discuss the main components and output electrical performance of various nanogenerators applied to the medical sector. Finally, the challenges and perspectives of the design, materials and fabrication process, signal processing, and reliability of nanogenerators are included.

## 1. Introduction

Due to the health problems caused by COVID-19, the World Health Organization (OMS) has implemented strategies for monitoring, controlling, and treating chronically ill patients [1,2]. An interesting strategy is digital home hospitalization, which could be used in future health contingencies [3,4,5]. This strategy is justified by the lack of hospital beds and the increase in the world population of older adults [6,7]. The world population could reach 8.5 billion and 9.7 billion in 2030 and 2050, respectively [8]. In addition, digital home hospitalization is suitable for people with chronic diseases who live in isolated areas [9,10]. Thus, patients could be cared for from their homes, preserving their integrity with the help of the implementation of a wireless patient monitoring tool.

In recent years, the internet of medical things (IoMT) and the internet of things (IoT) have registered great interest from the scientific and medical communities. The IoMT is a subarea developed and applied within the IoT to focus on different current medical applications. This process uses the acquisition, processing, transmission, and storage of medical information through specific devices and the security of patient data [11,12,13,14,15]. The IoMT processes and contains patients’ confidential data. Thus, a critical challenge of the IoMT is achieving data security [16,17]. The IoMT requires devices with soft, elastic, and biocompatible materials, safe performance, and stable electrical response [18,19,20]. In addition, these devices require a great capacity for monitoring different patient signals using electrical energy sources of small size and long lifetime. These devices must provide real-time data on the health status of patients, informing the patients, family members, medical centers, or doctors. In addition, the IoMT devices must alert possible health risks of patients.

Wireless and portable electronics have increased with the gradual technological development of 5th generation mobile networks around the world [21,22,23,24,25]. Using portable electronic devices and wireless connections, the IoMT technology may allow the possibility to control and assist patients. Thus, this technology can help patients’ health by providing and controlling medical treatments [26,27,28,29]. However, the future IoMT portable devices will require energy sources that replace conventional batteries, which have limitations such as limited lifetimes, large volume and weight, and toxic materials that can contaminate the environment. To improve the performance and lifetime of energy storage devices, recent investigations [30,31,32,33,34,35] have developed rechargeable metal-air batteries and metal-ion batteries, as well as supercapacitors. Another alternative to substitute conventional batteries is the fabrication of nanogenerators, which can harvest green energy from the environment or the human body. The nanogenerators offer advantages such as a long lifetime, small volume, light weight, a non-expensive fabrication process, and high output power density [36,37,38,39,40]. The nanogenerators can use the triboelectric and piezoelectric effects for harvesting biomechanical energy into electricity [41,42,43,44,45,46,47,48,49]. The piezoelectric nanogenerators can convert the mechanical deformations of the piezoelectric materials used in their structures into electrical voltages. On the other hand, triboelectric nanogenerators use both contact electrification and electrostatic induction between two moving triboelectric layers to generate an electrical potential difference. Table 1 depicts several advantages and disadvantages of piezoelectric and triboelectric nanogenerators, metal-air batteries, and supercapacitors.

**Figure 1:**
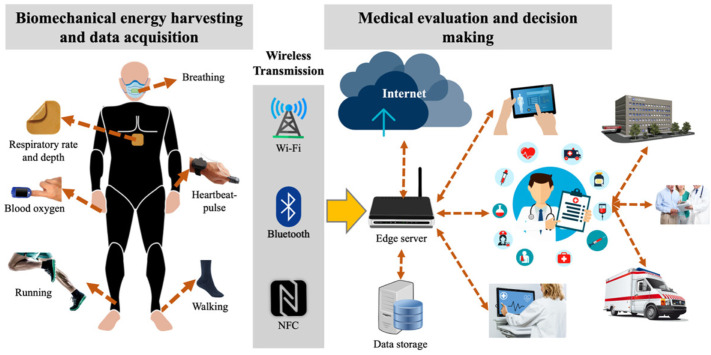
Potential application of piezoelectric and triboelectric nanogenerators to convert biomechanical energy into electrical energy, which could be used to power IoMT electronic devices.

Nanogenerators can harvest biomechanical energy due to the movement of the hands and fingers [50,51,52,53,54,55,56,57], wrists [58,59,60,61,62,63,64,65,66], knee joints [67,68,69,70,71,72,73,74], and feet [75,76,77,78,79,80,81,82,83,84]. In addition, this energy is generated by external agents, including the movement or friction of clothing, backpacks, and the airflow interacting with the human body when running, riding a motorcycle or bicycle, skating, and so on. Nanogenerators could also power future implantable medical devices. Implantable medical devices can maintain and monitor vital signs of patients, such as heart rate monitors, pacemakers, defibrillators, and brain stimulation [85,86,87]. However, these devices are still powered using conventional batteries, which have limited-service life. Thus, nanogenerators could harvest the biomechanical energy of patients for powering implantable medical devices [88,89,90,91,92,93,94,95,96,97,98,99,100,101,102]. Figure 1 illustrates the application of nanogenerators to convert biomechanical motion into electrical energy to power potential IoMT electronic devices.

We reviewed piezoelectric and triboelectric nanogenerators that can harvest green energy from environmental sources such as human body motion, mechanical vibrations, and wind. The harvested energy can be converted into electrical energy to power potential medical devices that monitor and process signals of different health parameters of patients. These signals could be transmitted to hospitals or medical centers using the IoMT. The operating principle, materials, and manufacturing processes of nanogenerators are discussed. Future medical applications of piezoelectric and triboelectric nanogenerators are reported, including materials, signal processing components, and packaging. Finally, we proposed the challenges and perspectives on the output performance of nanogenerators, taking into account the topics of design, materials and fabrication processes, signal processing, and reliability.

## 2. Operating Principle, Materials, and Fabrication

Triboelectric and piezoelectric nanogenerators have potential applications in the medical sector due to their high electrical output performance, simple structure and operation principle, and cost-effective fabrication process.

**Figure 2:**
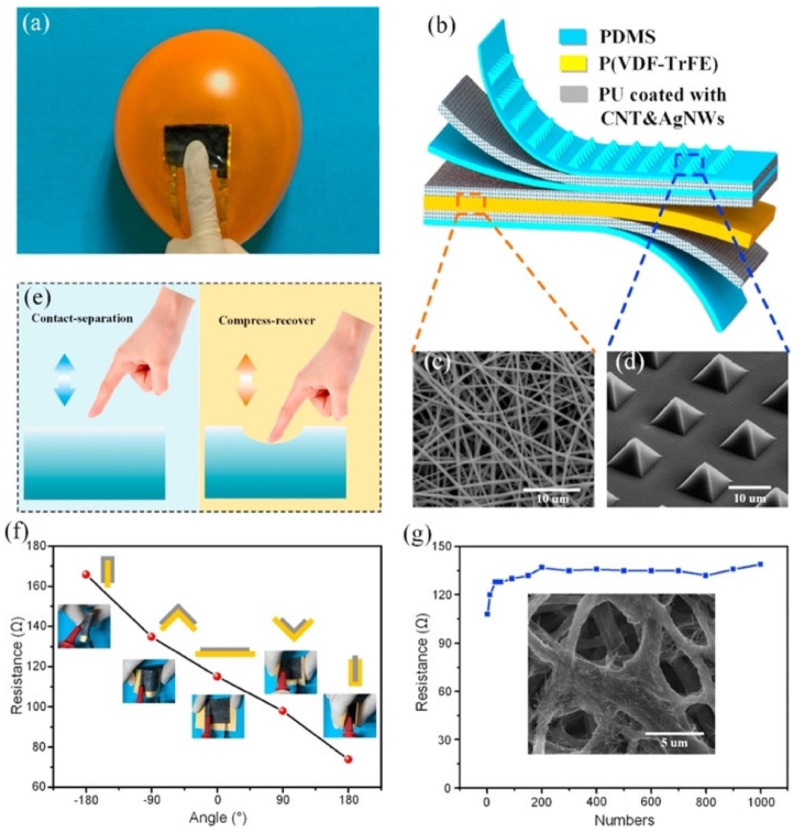
Flexible hybrid nanogenerator reported by Chen et al. [103]. (**a**) Nanogenerator placed on the surface of a balloon. (**b**) Schematic view of the different materials of the nanogenerator. (**c**) SEM image of the electrospun P(VDF-TrFE) nanofiber mat. (**d**) SEM image of the micro-patterned PDMS layer. (**e**) Process of contact-separation and compress-recover on a soft surface. (**f**) Measurements of resistance of the conductive fiber mat as a function of its bending angle. (**g**) Stability results on the resistance variation of the fiber-based electrode under fold/unfold deformation for 1000 cycles. SEM image of the fiber-based electrode. Reprinted with permission from [103]. Copyright ©2017, Elsevier Ltd.

**Figure 3:**
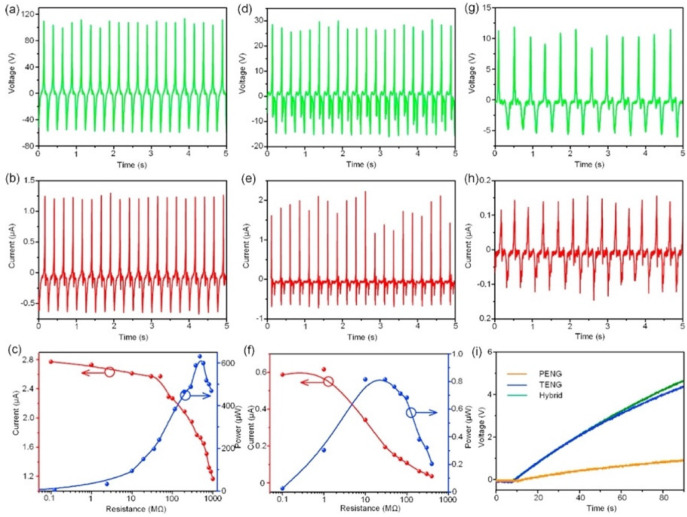
Electrical output performance of the flexible hybrid nanogenerator reported by Chen et al. [103]. (**a**) The output voltage, (**b**) current, and (**c**) maximum peak power as a function of resistance load of the TENG module. (**d**) The output voltage, (**e**) current, and (**f**) maximum peak power versus resistance load of the PENG module under compression. (**g**) The output voltage and (**h**) current of the PENG module under bending mode. (**i**) The charge voltage of a capacitor of 1 μF using the hybrid nanogenerator. Reprinted with permission from [103]. Copyright ©2017, Elsevier Ltd.

**Figure 4:**
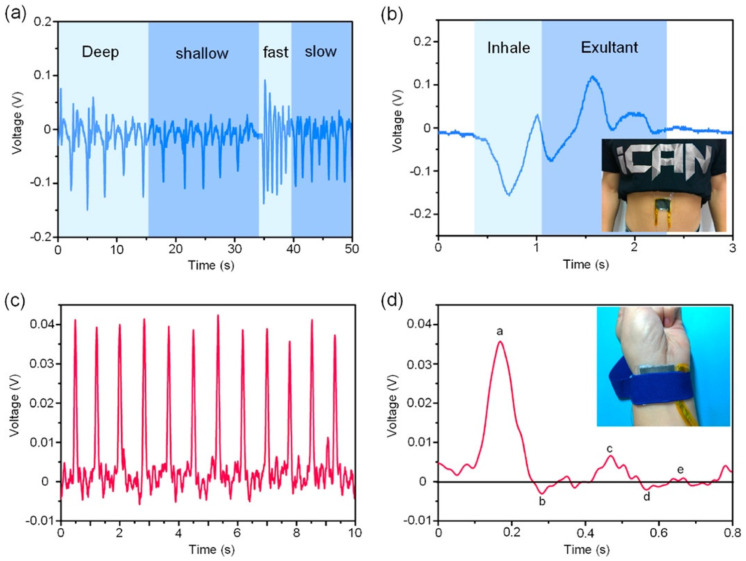
Results of real-time physiological monitoring using the flexible hybrid nanogenerator developed by Chen et al. [103]. (**a**) Respiration signal during 50 s with four different breathing modes such as deep, shallow, fast, and slow. (**b**) The enlarged signal in one breath cycle of a patient, which was measured using the hybrid nanogenerator attached to the belly. (**c**) The real-time artery pulse signal and (**d**) its enlarged signal of output voltage in one cycle that was measured with the hybrid nanogenerator. Reprinted with permission from [103]. Copyright ©2017, Elsevier Ltd.

In 2017, Chen et al. [103] reported a flexible hybrid nanogenerator that can be attached on soft surfaces, such as human skin, for body motion harvesting and monitoring physiological signals. A single-electrode TENG and a PENG are integrated into the nanogenerator to improve its electromechanical performance (Figure 2). The PENG module used poly(vinylidenefluoride-co-trifluoroethylene) P(VDF-TrFE) nanofiber mats as the piezoelectric structure, and polydimethylsiloxane (PDMS) as the protective layer. Thermoplastic polyurethane (PU) nanofibers coated with carbon nanotubes (CNT) and silver nanowires (AgNWs) were employed in the electrodes. Thus, the PU electrodes sandwiched the piezoelectric layer. The PENG part was separated from the TENG part by a PDMS isolation film to produce piezoelectric and triboelectric outputs. Another PDMS layer operated as triboelectric material, and the PU film as an electrode for the single-electrode TENG. Based on the triboelectric and piezoelectric mechanisms, this hybrid nanogenerator registered a maximum peak power up to 84 mW/m^2^ and 0.11 mW/m^2^ for the TENG and PENG modules under compressive stress (Figure 3). This nanogenerator can be attached to any soft hybrid to convert tapping and pressing energy into electricity. The hybrid nanogenerator can be placed on the skin for real-time monitoring of human physiological signals, such as respiratory information and radial artery pulse. Figure 4 shows the hybrid nanogenerator attached to the back of the hand to collect the body motion energy of closing the fist and punching. This nanogenerator can be mounted on the abdomen for monitoring the human respiratory rate and depth. Thus, the nanogenerator could be employed for monitoring the respiratory condition of patients. Also, this nanogenerator has potential applications for self-powered healthcare monitoring systems and e-skins.

**Figure 5:**
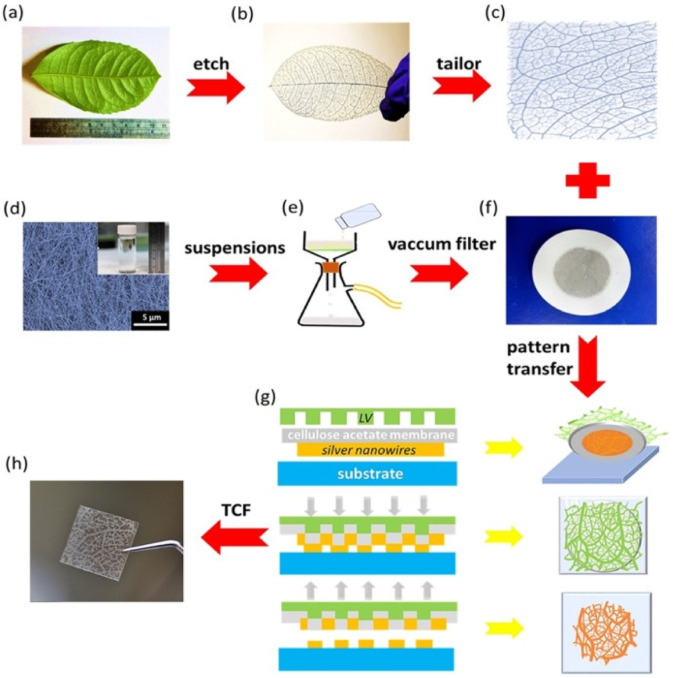
Network of LV-like Ag nanowires of the hybrid nanogenerator fabricated by Sun et al. [104]. Images of (**a**) the structure of a ramified leaf used as a mold to fabricate the patterns of TEs. (**b**) The skeleton of the leaf venation and (**c**) LV network were prepared in a square shape to be used as a mold. (**d**) SEM image of Ag nanowires. These nanowires were synthesized in a small bottle. (**e**) Schematic of vacuum filtration of the Ag nanowires. (**f**) Image of Ag nanowires film filtered using a cellulose acetate membrane. (**g**) Schematic of a modified dry transfer printing technique to fabricate the LV-like Ag nanowires network. (**h**) Image of the LV-like Ag network on a PDMS substrate (size of 3.5 cm × 3 cm). Reprinted with permission from [105]. Copyright ©2018, Elsevier Ltd.

**Figure 6:**
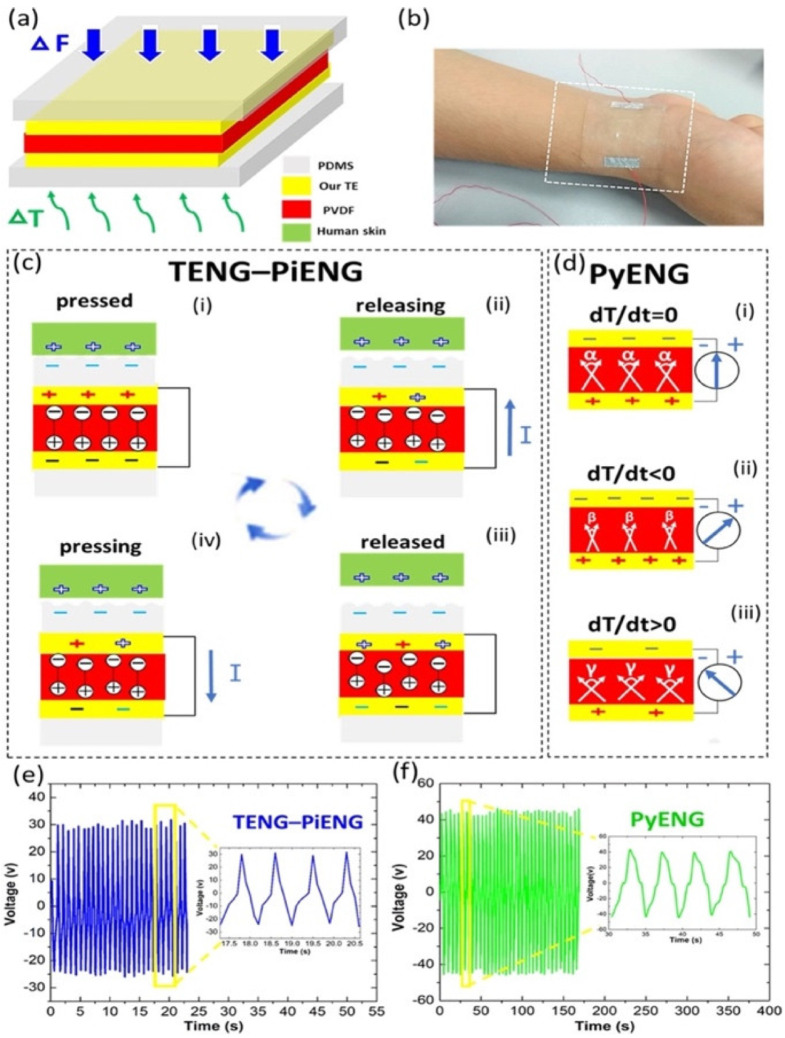
Working mechanism of the flexible hybrid nanogenerator designed by Sun et al. [104]. (**a**) Schematic view of the components of the hybrid nanogenerator. (**b**) Image of the hybrid nanogenerator placed on the wrist. Working mechanism of the (**c**) TENG-PiENG and (**d**) PyENG of the hybrid nanogenerator. Open-circuit voltage of the (**e**) TENG-PiENG and (**f**) PyENG. Reprinted with permission from [104]. Copyright ©2018, Elsevier Ltd.

**Figure 7:**
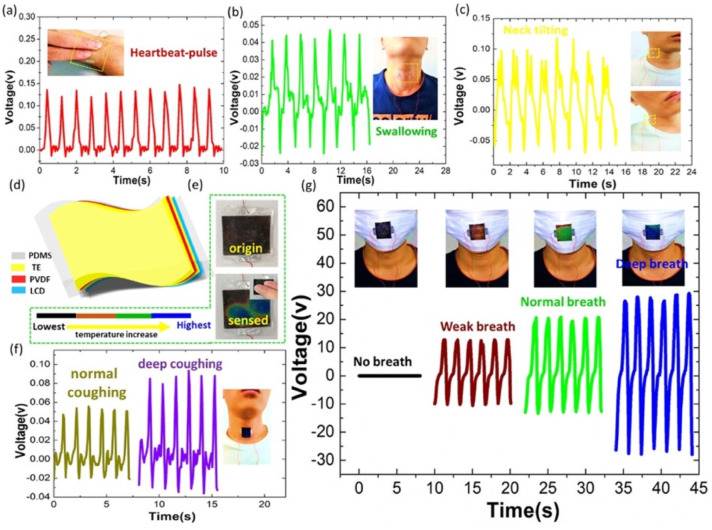
Potential applications of the flexible hybrid nanogenerator reported by Sun et al. [104]. Voltage output of the hybrid nanogenerator for assessing (**a**) heartbeat pulse, (**b**) swallowing, and (**c**) neck tilting. (**d**) Schematic view of a smart device composed of transparent hybrid nanogenerator and LCD film. (**e**) Smart device for temperature monitoring. (**f**) Voltage output of the smart device for sensing different degrees of coughing. (**g**) Voltage output of the smart device for monitoring three types of breathing conditions. Reprinted with permission from [104]. Copyright ©2018, Elsevier Ltd.

In 2018, Sun et al. [104] designed a biocompatible triboelectric–piezoelectric–pyroelectric hybrid nanogenerator with high flexibility. This nanogenerator has a transparent structure formed by polyvinylidene fluoride (PVDF), PDMS, and Ag nanowires. The PVDF material acts as piezoelectric and pyroelectric film and the PDMS acts as a triboelectric film. The Ag nanowires are used as high-performance transparent electrodes (TEs) and can be distributed into a network like a leaf venation (LV), inspired by optimized LV (Figure 5). Thus, the working mechanism of the hybrid nanogenerator is integrated by a triboelectric nanogenerator, a piezoelectric nanogenerator (TENG-PiENG), and a pyroelectric nanogenerator (pyENG). This nanogenerator can be mounted on different body parts to collect mechanical and thermal energy from the human body. This nanogenerator has an environmentally friendly design, which the structure of LV can reuse many times. The nanogenerator structure registered a minimum sheet resistance of 1.4 Ωsq^−1^ with 82% transmission and sheet resistance of 68.2 Ωsq^−1^ with an ultra-high transmission of up to 99%. The hybrid nanogenerator can harvest a maximum open-circuit output voltage of 55 V and 86 V from mechanical and thermal energy with the three coupled transduction mechanisms (Figure 6). The nanogenerator has a potential application for assessing the patient’s health status with a cold, using frequency, coughing, and breathing through the amplitude of the voltage (Figure 7). The hybrid nanogenerator can monitor various human physiological signals, such as heartbeat, swallowing, and neck tilting. This nanogenerator could be used for cost-effective medical diagnostics and prognosis of cardiovascular, Parkinson, and esophagus diseases. Also, the nanogenerator integrated with a thermochromic liquid crystal display (LCD) could operate as a thermometer for medical diagnostics.

**Figure 8:**
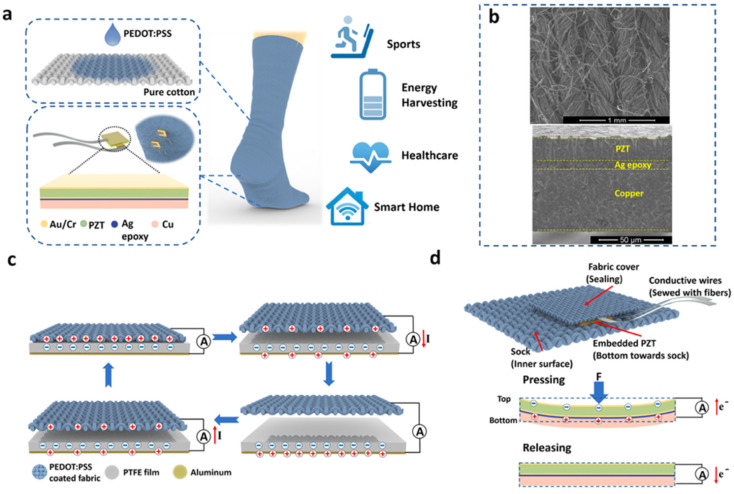
TENG and piezoelectric device integrated in a S^2^-sock manufactured by Zhu et al. [105]. (**a**) Schematic of the materials used in the triboelectric S^2^-sock coupled with piezoelectric devices for various applications, including energy harvesting, healthcare, smart home, and sports. (**b**) (Top) SEM image (top view) of PEDOT:PSS-coated textile. SEM image of a cross-section view of piezoelectric device. (**c**) Working mechanism of TENG sock using contact-separation condition. (**d**) Working mechanism of piezoelectric device and integration design for S^2^-sock. Reprinted with permission from [105]. Copyright ©2019, American Chemical Society.

**Figure 9:**
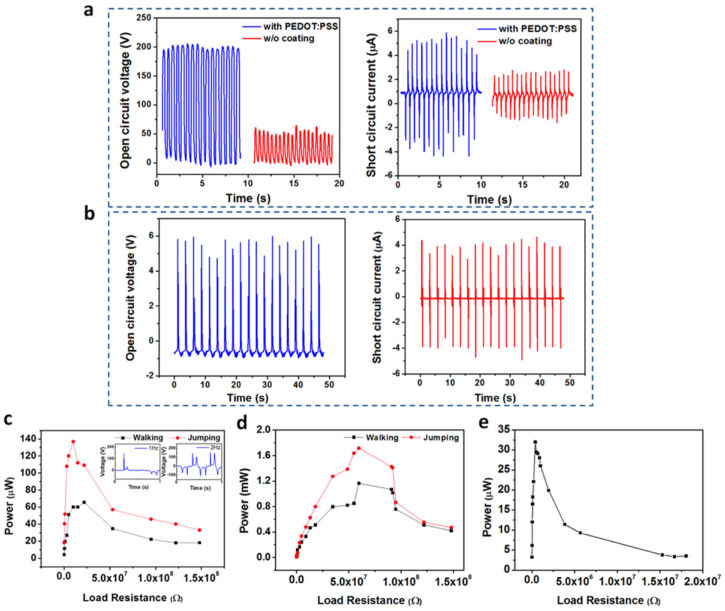
Output electrical performance of the TENG and piezoelectric device integrated in a S^2^-sock designed by Zhu et al. [105]. (**a**) Results of open-circuit voltage and short circuit current of the sock with and without PEDOT:PSS under contact-separation operation with respect to PFTE film placed on the ground (Figure 9c). (**b**) Results of open-circuit voltage and short-circuit current of piezoelectric device integrated to the sock (Figure 9d). (**c**,**d**) Output power as a function of the external load resistance with and without shoes. (**e**) Output power of the piezoelectric device as a function of external load resistance. Reprinted with permission from [105]. Copyright ©2019, American Chemical Society.

In 2019, Zhu et al. [105] investigated a self-powered and self-functional cotton sock (S^2^-sock) based on a poly(3,4-ethylenedioxythiophene) polystyrene sulfonate (PEDOT:PSS)-coated fabric TENG and lead zirconate titanate (PZT) piezoelectric device (Figure 8). The PEDOT:PSS is a polymer with high conductivity and mechanical stability. A Cu substrate was bonded to the piezoelectric device to improve the ductility and optimize the mechanical behavior. This sock can produce the featured waveforms for walking pattern recognition and motion tracking of patients under home care. The experimental tests show that in-shoe situation, the TENG part of the sock can generate the output power of 66 μW and 137 μW at 1 Hz walking and 2 Hz jumping with a load resistance of 10 MΩ, respectively (Figure 9). For indoor use, the TENG part of the sock stepping on a polytetrafluoroethylene (PTFE) film, which is mounted on the ground, registers maximum powers of 1.17 and 1.71 mW under a load resistance of 59.7 MΩ. This allows a power density of 11 μW/cm^2^ considering a contact area close to 150 cm^2^. For the piezoelectric device (size of 5 cm × 5 cm), the maximum output power was 32 μW under load resistance of 0.4 MΩ and 12 N at 1 Hz. The maximum power density of the PZT device is 128 μW/cm^2^, which offers a good performance for smart textiles. For the indoor case using bare shocks at home, the pattern recognition and motion tracking can be measured for home care applications of the IoMT. The S^2^-shock was used to measure the mimetic motions of a Parkinson’s disease patient regarding normal motion, loss of stride, and freezing of gait (FOG). Moreover, this sock can collect energy from body motion and sense diversified physiological signals for healthcare and home sports. In addition, this cotton sock can be enhanced by incorporating wireless transmission modules and integrated circuits.

In 2019, Li et al. [106] reported a hybrid nanogenerator based on cellulose nanofibril (CNF) as a triboelectric layer and bacterial cellulose (BC) with nanoparticles of BaTiO_3_ and a multi-walled carbon nanotube (MWCNT) as piezoelectric layer. The CNF fibers were treated by employing a mixed solution of HNO_3_ and H_2_SO_4_. In addition, a -NO_3_ group is applied to enhance the electronegativity of the CNF fibers. This hybrid nanogenerator is formed by two composite layers. The top arched layer acts as PENG, using a functionalized BC paper with two Ni electrode films. The PENG has an arched shape to improve the strength of the effective strain applied to the bacterial cellulose film. The bottom layer operates as TENG integrated by a Nitro-CNF paper and a bottom Cu electrode film. The working mechanism of the PENG consists of the strain of the piezoelectric layer caused by external forces on its top surface, which generates a voltage variation between the two Ni electrodes. On the other hand, TENG operates with contact electrification and electrostatic induction. The triboelectric and piezoelectric parts have high output electrical performance, achieving short-circuit current density and open-circuit voltage of 1.23 μA/cm^2^, 37 V, 220 nA/cm^2^, and 22 V, respectively. With a rectifier circuit to integrate the outputs of both triboelectric and piezoelectric parts, the hybrid nanogenerator has a short-circuit current density and open-circuit voltage of 1.6 μA/cm^2^ and 18 V, respectively. This cellulose-composed nanogenerator has an environmentally friendly simple structure, light weight, and cost-effective fabrication. Thus, this nanogenerator could apply to wearable or implantable devices and self-powered electronic sensors.

In 2020, Syu et al. [107] reported a biomimetic and flexible hybrid self-powered sensor (BHSS) that was formed by triboelectric and piezoelectric elements. The triboelectric structure is based on the shell of Mytilidae and is fabricated with PDMS film. The piezoelectric component contains porous PVDF fibers deposited on a printed circuit board (PCB) substrate. To develop the biomimetic Mytilidae surface, Mytilidae nano-structured patterns on a PDMS layer were applied using a soft transfer molding process. This hybrid sensor registered an open-circuit voltage and short-circuit current of 15 V and 115 nA, respectively. For a load resistance of 10 MΩ, the hybrid sensor achieved a maximum average power density of 675 μW/m^2^. The hybrid sensor was tested. This self-powered sensor was attached on the thumb, index, and middle fingers of a latex glove for monitoring bottle-holding actions. For this experiment, its electrical signals under three plastic bottles with different water volumes (loading weight of 0.65 kg, 1.3 kg, and 2.06) were examined. The electrical signals of the sensors were studied for various contact positions along the bottles. This self-powered sensor could be used for wearable electronic devices and monitoring biomechanical motion, and human gesture recognition, using a machine learning algorithm of long short-term memory.

**Figure 10:**
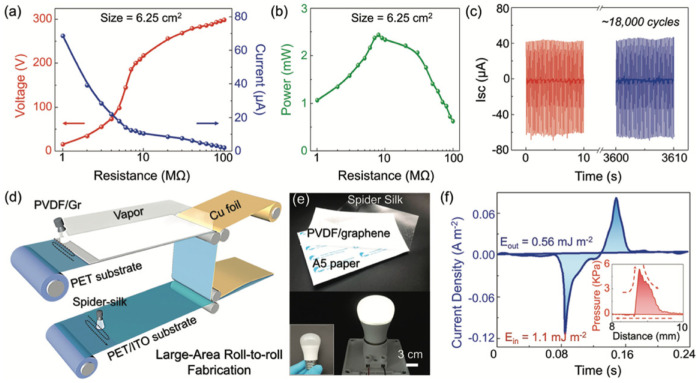
Performance of the hybrid nanogenerator developed by Huang et al. [108]. (**a**) Output open-circuit voltage and short-circuit current of the hybrid nanogenerator as a function of load resistance. (**b**) The output power of the hybrid nanogenerator as a function of load resistance. (**c**) Results of stability and durability tests of the hybrid nanogenerator. Short-circuit current measured over 18,000 cycles. (**d**) Schematic of the prototype for the large-scale fabrication of the hybrid nanogenerator. (**e**) Photographs of the spider silk and PVDF/graphene films (both films with area equal to A5 paper), and white LED light (3 W). This LED was lightened using a hybrid nanogenerator with an area of 48 cm^2^. (**f**) The output current density of the hybrid nanogenerator during one cycle of hand tapping. The inset illustrates the input pressure generated by hand-tapping under a distance range. Reprinted with permission from [108]. Copyright ©2020, WILEY-VCH Verlag GmbH & Co. KGaA, Weinheim.

**Figure 11:**
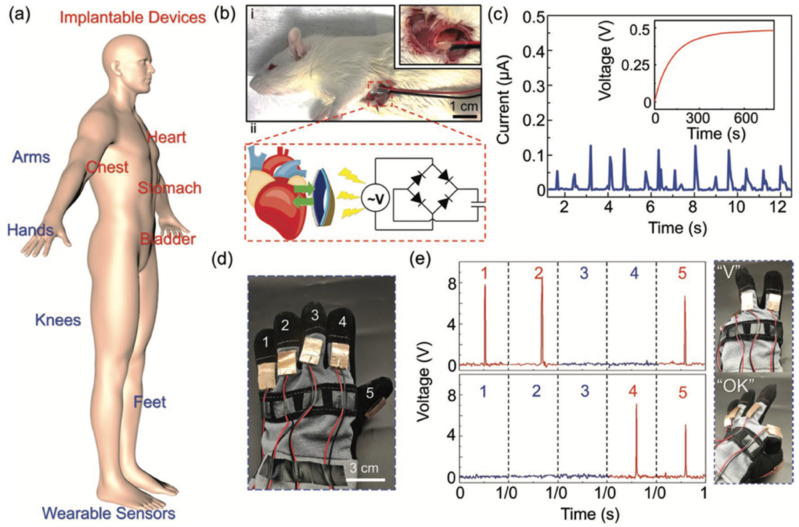
Potential applications of the hybrid nanogenerator proposed by Huang et al. [108]. (**a**) Schematic of a hybrid nanogenerator with potential applications in implantable devices and wearable sensors. (**b**) (i) Photograph of a hybrid nanogenerator placed in the subdermal chest of a Sprague-Dawley rat; (ii) Schematic of the working mechanism of the nanogenerator for energy harvesting from a beating rat heart. For this, a rectifier circuit and a capacitor (4.7 μF) were implemented. (**c**) Current signals of an implanted hybrid nanogenerator related to the beating heart. Inset shows the storage voltage of a capacitor (4.7 μF). (**d**) Photograph of a hybrid nanogenerator adhered on the glove for hand-gesture monitoring. (**e**) Output voltage signals of nanogenerators placed on the five fingers for “Victory” and “OK” hand gestures. Reprinted with permission from [108]. Copyright ©2020, WILEY-VCH Verlag GmbH & Co. KGaA, Weinheim.

Huang et al. [108] fabricated a flexible biocompatible triboelectric-piezoelectric nanogenerator by employing recombinant spider silk and poly(ethylene terephthalate) (PET)/PVDF films. Graphene was incorporated into PVDF to improve the piezo-PVDF performance. Thus, the PET/PVDF-graphene composite film was added with genetically engineered spider silk. The electron-cloud-potential-well model was used to describe the charge transfer between spider silk and PET, with or without the PVDF film. The electrons in molecular orbits of spider silk protein can be transferred to the empty orbits of PET film. This transference of electrons depends on the difference in potential-well depths (difference of the surface electron potential) between both materials. PVDF, under mechanical strain, can alter the surface electron potential of the coupling material. Due to this, PVDF was added to PET film to enhance its surface potential. Thus, the improved surface-potential difference between PET/PVDF and spider silk can increase their electrons transfer and amount of harvested energy. Figure 10 depicts the output electrical signals and stability results of the hybrid nanogenerator. This hybrid nanogenerator (area of 6.25 cm^2^) registered a maximum output power density of 4016 mW/m^2^ under a load resistance of 8 MΩ. For this resistance, the nanogenerator reached the output voltage, output short-circuit current, and output power close to 200 V, 12 μA, and 2.51 mW, respectively. This nanogenerator could be used as wearable hand-gesture sensors, body motion sensors, bioenergy harvesters, and implantable organ monitors (Figure 11). For instance, this nanogenerator could be included in implantable sensors for monitoring signals of the heart, stomach, chest, and bladder.

**Figure 12:**
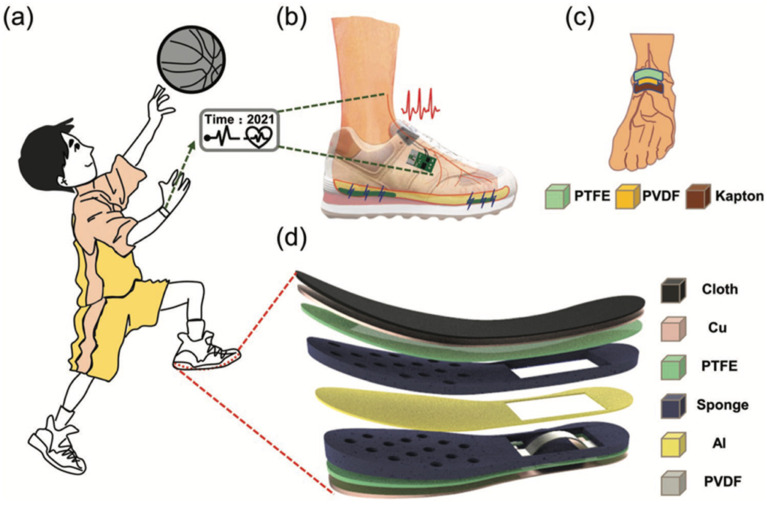
Schematic of the main components of the isolate hybrid nanogenerator presented by Du et al. [109]. (**a**) IHN applied to a sports shoe for monitoring the footstep types. (**b**) Schematic of the self-powered system for dorsalis pedis artery monitoring. (**c**) Schematic of the signal acquisition of the dorsalis pedis artery sensor. (**d**) Schematic of the materials and elements for the TENG and PENG modules of the IHN. Reprinted with permission from [109]. Copyright ©2022, John Wiley and Sons.

In 2022, Du et al. [109] designed a shoe insole hybrid nanogenerator (IHN) composed of a multilayered TENG and arched PENG. This nanogenerator can convert the mechanical energy of footsteps into electrical energy. Moreover, this nanogenerator can identify three types of motion states: walking, stepping, and jumping. This nanogenerator has high performance of electrical stability and durability, which is suitable to collect biomechanical energy of feet to power wearable electronic sensors. In addition, this nanogenerator has potential application for self-powered biosensor systems in the fields of sports and medicine. Figure 12 illustrates an application of the IHN integrated with a dorsalis pedis artery sensor and a processor module. The sensor is placed inside the tongue of the shoe and the processor module is mounted on the outside of the shoe. The sensor contains a PVDF film that is packaged with PTFE and Kapton films. The PVDF film harvests the vibration signal of the pulse and converts it into an electrical signal. The hindfoot and forefoot of the IHN are formed by the arched PENG and TENG, respectively. The TENG part has three triboelectric films (PTFE-Al-PTFE), in which two sponge layers with through-holes are added to separate the Al sheet from both PTFE films. Both PTFE films and the Al layer are contacted and separated due to the motion (e.g., walking, stepping, and jumping) of the foot. On the back of PTFE is attached the Cu electrode that operates as a source for the charge transference caused by electrostatic induction. In addition, the Al sheet can act as both an electrode and a triboelectric layer. On the other hand, a surface area (2 cm × 7 cm) of the sponge is extracted to incorporate the arched PENG. This PENG is composed of a PVDF film that is packaged by an arched PE and Kapton film. Furthermore, the IHN was encapsulated using a cotton cloth and PE film, which avoids the effect of sweat on the electrical performance of the hybrid nanogenerator.

**Figure 13:**
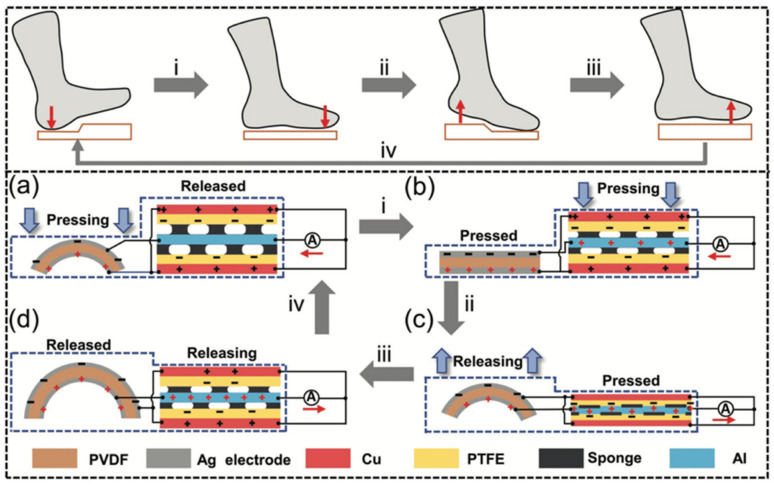
Working mechanisms of the PENG and TENG parts of the IHN during one walking cycle [109]. Schematic of the charge transfer process of the PENG and TENG parts when (**a**) the hindfoot falls, (**b**) the forefoot falls, (**c**) the hindfoot lifts, and (**d**) the forefoot lifts, respectively. Reprinted with permission from [109]. Copyright ©2022, John Wiley and Sons.

**Figure 14:**
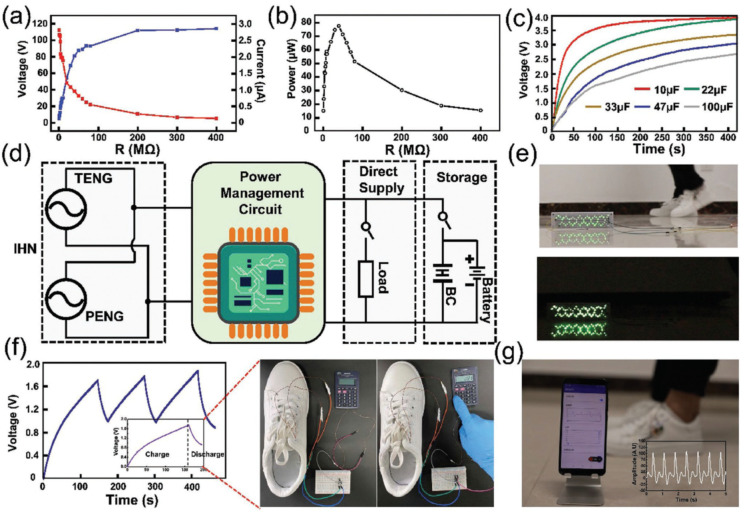
Electrical output performance and applications of IHN proposed by Du et al. [109]. (**a**) Output voltage and current as a function of external load resistance. (**b**) Output power as a function of external load resistance. (**c**) Charge response of four capacitors powered by IHN. (**d**) Schematic view of the electrical circuit for the IHN direct power supply and energy storage system. (**e**) LEDs lighted using the IHN. (**f**) Output voltage and calculator powered by IHN. (**g**) Pulse signal of the dorsalis pedis monitoring system, which was sent to a mobile phone. Reprinted with permission from [109]. Copyright ©2020, WILEY-VCH Verlag GmbH & Co. KGaA, Weinheim.

Figure 13 shows the working mechanism of the IHM reported by Du et al. [109] during a walking cycle. Due to the pressure of the heel of the shoe in a walking cycle, the PENG shape is altered from arch to plane. This deformation generates a potential difference between both electrodes of the PENG. When the forefoot falls on the ground, the PTFE and Al layers are contacted in the through-hole of the sponge. The increment of the pressure of the forefoot increases the contact area between both PTFE and A layers. After the contact of both layers, a surface charge transfer induces between them a potential difference in the back Cu electrode (Figure 13b). This potential difference generates the electrons flow from the Cu electrode to the Al electrode using the external circuit. The PENG returns to arch shape after the heel lifts, generating a current opposite to that when the hindfoot falls (Figure 13c). When the forefoot is lifted, the PTFE and Al layers of the TENG are separated. This causes the electrons flow from the Al electrode to the Cu electrode in the external circuit, which has an opposite direction to that when the forefoot falls (Figure 13d). This sequence of four states occurs during one cycle of walking. Due to the parallel design of TENG and PENG, both nanogenerators have the same electron flow direction. It allows the composite signal output of the IHN to be higher than those of the TENG and PENG. From human body motions, the IHN generated a maximum open circuit voltage of 150 V and a short-circuit current of 4.5 μA, respectively. Figure 14g shows the pulse signal of a dorsal pedis monitoring system that is supplied by the IHN.

## 3. Potential Applications

This section describes the performance of potential biomedical applications of triboelectric or piezoelectric nanogenerators.

**Figure 15:**
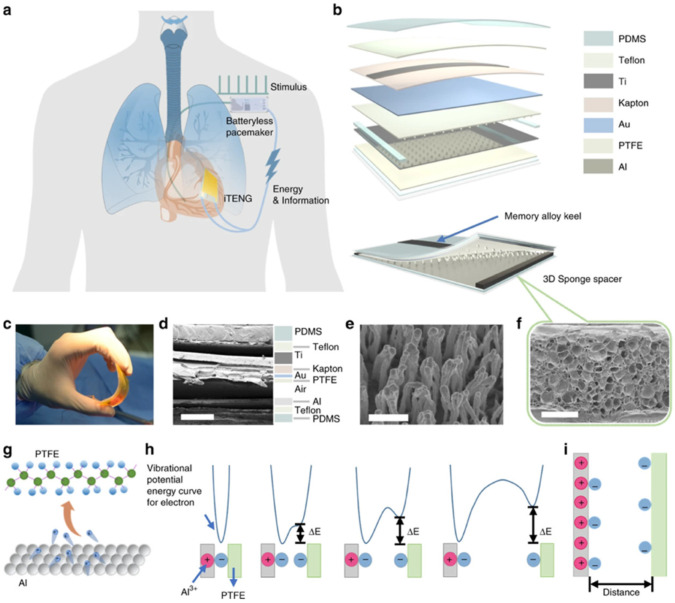
Schematic view of main components of symbiotic pacemaker system developed by Ouyang et al. [110]. (**a**) Schematic representation of different elements used in the symbiotic cardiac pacemaker system. (**b**) Illustration of the materials used in the iTENG. (**c**) Image of the iTENG structure under bending stress. (**d**) SEM image of different layers of an iTENG section (scale bar: 500 mm). (**e**) SEM image of the nanostructure on the PTFE layer (scale bar: 1 mm) of the iTENG. (**f**) SEM image of a 3D elastic sponge structure (scale bar: 500 mm). (**g**,**h**) Schematic view of the mechanism of charge transfer. (**i**) Model of the charge separation caused by the charge transfer. Reprinted with permission from [110]. Copyright ©2019, Springer Nature.

**Figure 16:**
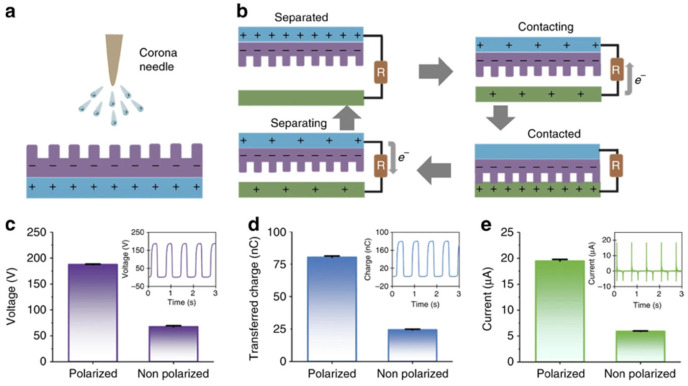
Working mechanism of the PTFE-based iTENG designed by Ouyang et al. [110]. (**a**) Schematic view of a corona discharge system. (**b**) Working mechanism of the iTENG. (**c**–**e**) Results of electrical performance (output voltage, transferred charge, and current) of the polarized and non-polarized PTFE film considering a linear motor. Reprinted with permission from [110]. Copyright ©2019, Springer Nature.

**Figure 17:**
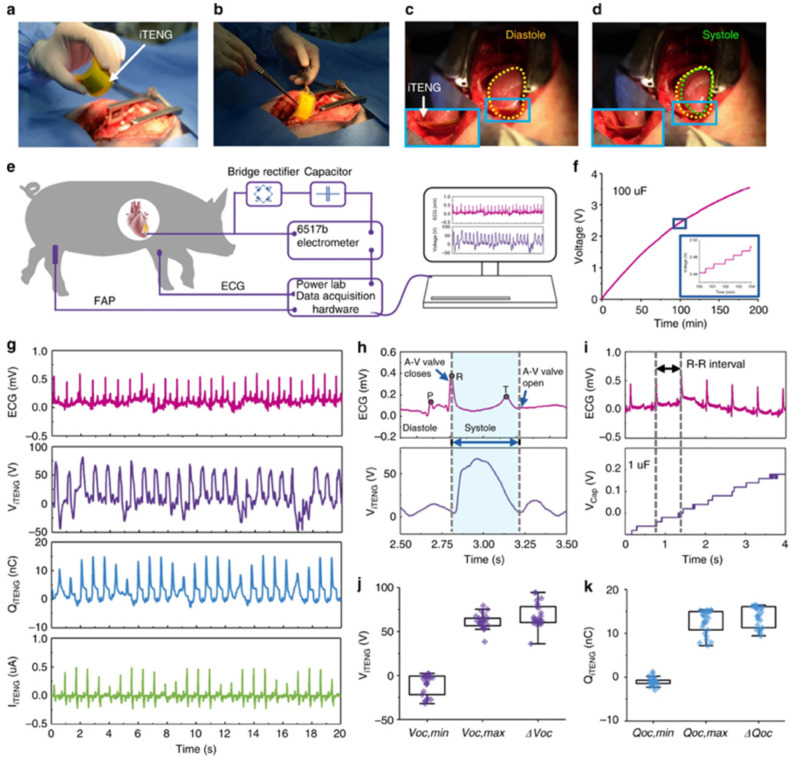
Electrical performance of the iTENG implanted in an animal specimen [110]. (**a**,**b**) Photographs of the iTENG implanted between the heart and pericardium of a Yorkshire porcine. (**c**,**d**) Photographs of the iTENG driven by the cardiac motion of a Yorkshire porcine. (**e**) Schematic view of the electrical performance test of the iTENG. (**f**) Charging response of a capacitor (100 μF) that is charged by iTENG. (**g**) In vivo electrical tests of the short-circuit current, transferred charge, and open-circuit voltage of the iTENG, as well as simultaneously recorded ECG signals. (**h**) Output open-circuit voltage of the iTENG and simultaneously recorded ECG results. (**i**) Charging signal of a capacitor (1 μF) charged by iTENG and ECG curve. (**j**) Statistical results of voltage shift, maximum voltage, and minimum voltage of the iTENG. (**k**) Statistical results of the transferred charge shift, maximum transferred charge, and minimum transferred charge of the iTENG. Reprinted with permission from [110]. Copyright ©2019, Springer Nature.

**Figure 18:**
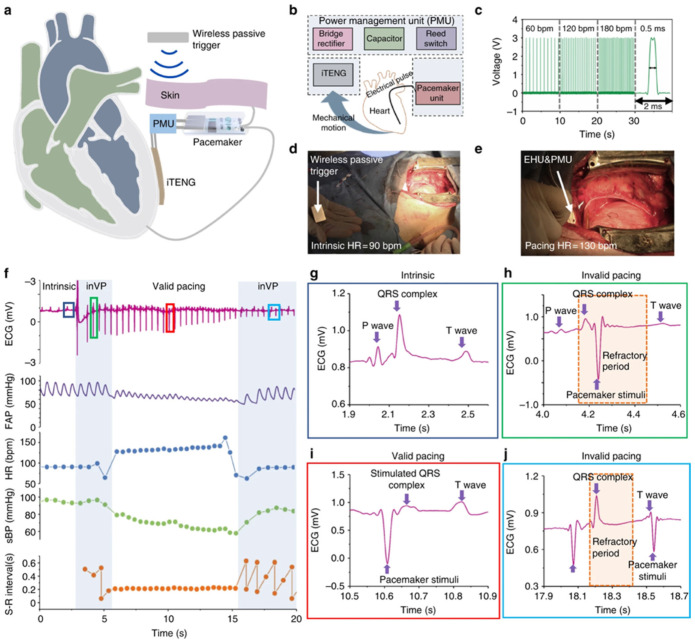
Performance of the symbiotic cardiac pacemaker designed by Ouyang et al. [110]. (**a**) Representation of the pacemaker powered by iTENG that harvests energy from the cardiac motion of a Yorkshire porcine. (**b**) Illustration of the main components of the symbiotic cardiac pacemaker system. (**c**) Stimulation voltage pulse under different frequencies produced by a pacemaker unit. (**d**,**e**) Photograph of the symbiotic cardiac pacemaker system switched on by wireless passive trigger in a pig. (**f**) Results of the stimulus-R wave intervals (S-R), systolic blood pressure (sBP), heart rate (HR), femoral artery pressure (FAP), and ECG during the stimulation stage of the symbiotic pacemaker unit. (**g**) ECG signal of the heart rate considering a normal systolic blood pressure. (**h**) ECG signal under a pacing stimulus in the refractory period, considering a normal systolic blood pressure. (**i**) ECG signal of the heart successfully placed by the symbiotic pacemaker unit, with decreased systolic blood pressure. (**j**) ECG signal under failed pacing stimuli, regarding a restored systolic blood pressure. Reprinted with permission from [110]. Copyright ©2019, Springer Nature.

The PENGs and TENGs can be used in the modulation of neuronal activity, diagnostics for cardiovascular treatment, heart implants, drug delivery, and abdominal implants, among others. Ouyang et al. [110] reported an implanted symbiotic pacemaker integrated by a triboelectric nanogenerator, a power management unit (PMU), and pacemaker unit. The implantable triboelectric nanogenerator (iTENG) can harvest and store energy from cardiac motion. The energy collected by the iTENG is stored in the capacitor of the PMU. Next, this stored energy can be used to drive the pacemaker unit, generating pacing electrical pulses, and controlling the rate of cardiac motion (Figure 15). Figure 16 shows the operating principle of the PTFE-based iTENG. Figure 17 depicts the electrical performance of the iTENG implanted in the chest of a Yorkshire porcine. For this case, the iTENG was located between the heart and pericardium of the pig. In addition, the PTFE component was placed on the left ventricular surface. Figure 18 illustrates the symbiotic cardiac pacemaker system in vivo using a wireless passive trigger. The pacemaker unit produces electrical pulses that can induce myocardial contraction and adjust the heart rate using pacing electrodes. The electrical pulses registered the output voltage and duration of 3 V and 0.5 ms, respectively. The symbiotic pacemaker could correct sinus arrhythmia and prevent damage. The iTENG achieved an open circuit voltage of 65.2 V and harvested energy from each cardiac motion cycle up to 0.495 mJ. This energy harvested is higher than that required for endocardial pacing (0.377 mJ). This iTENG could be employed for implantable medical electronic devices due to its high output power density and suitable stability. Thus, conventional batteries could be substituted.

**Figure 19:**
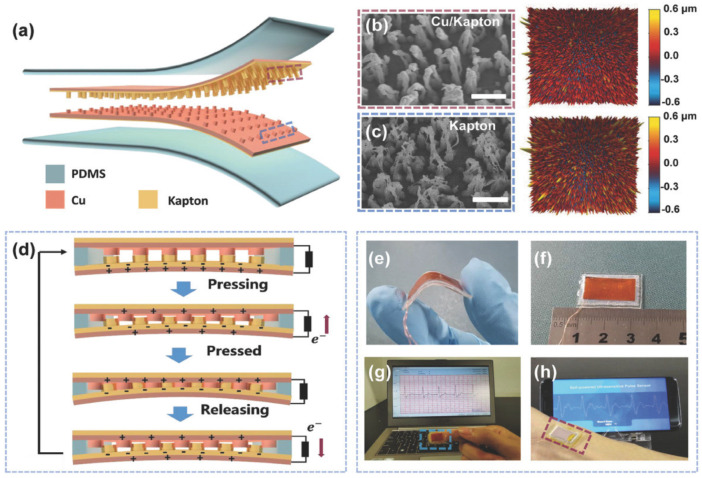
Components and operating mechanism of the SUPS developed by Ouyang et al. [111]. (**a**) Schematic view of the structure of the triboelectric nanogenerator of the SUPS. (**b**,**c**) SEM and atomic force microscopy (AFM) images of the nanostructured Cu and Kapton film, respectively. (**d**) Schematic view of the operating principle of the triboelectric nanogenerator. (**e**) Illustration of the good flexibility of the SUPS. (**f**) Image of the size of the SUPS structure (2 cm × 1 cm). (**g**,**h**) Experimental results of the signal outputs of SUPS pressed on the finger and radial artery region, respectively. Reprinted with permission from [111]. Copyright ©2017, WILEY-VCH Verlag GMbH & Co. KGaA, Weinheim.

**Figure 20:**
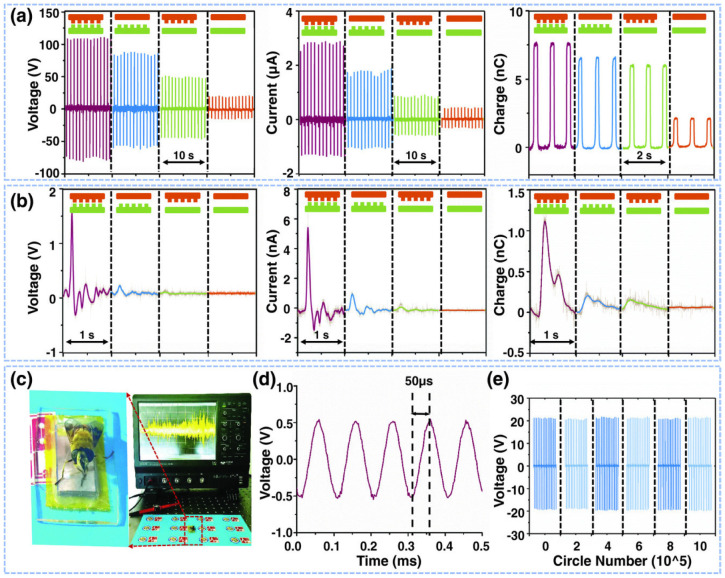
Electrical output performance of the SUPS with four different surface structures of the triboelectric films [111]. (**a**) Results of the output voltage, current, and transferred charge of SUPS when a lineal motor applied a compressive force of 50 N over four distinct structural configurations of triboelectric films. (**b**) Results of the output voltage, current, and transferred charge of SUPS placed on the radial artery and considering four different structural configurations of triboelectric films. (**c**) Real-time output voltage of SUPS caused by the oscillations (frequency close to 200 Hz) of the wings of a honeybee. (**d**) Output voltage of SUPS due to mechanical vibrations signals (10 kHz) generated by a loudspeaker. (**e**) Stability tests of the open-circuit voltage of SUPS during one million operating cycles. For this test, external forces of 30 N were applied on SUPS using a linear motor. Reprinted with permission from [111]. Copyright ©2017, WILEY-VCH Verlag GMbH & Co. KGaA, Weinheim.

Ouyang et al. [111] fabricated a flexible, self-powered, ultrasensitive pulse sensor (SUPS) formed by a triboelectric active sensor, which has an output voltage of 1.52 V, a peak signal–noise ratio of 45 dB, and good stability close to 107 cycles, as well as no expensive components. The SUPS structure (Figure 19) is composed of a nanostructured Kapton (n-Kapton) triboelectric film (100 mm thick) with an electrode Cu film (50 nm thick) deposited on its back side, a second Cu film (50 nm thick) deposited on other Kapton film to form a nanostructured Cu (n-Cu) film, and a spacer. These components are encapsulated using an elastomer. The working mechanism of the SUPS depends on the coupling of contact electrification and electrostatic induction (Figure 19d). The concave structures of the device allow the contact electrification and electrostatic induction to occur simultaneously. This characteristic of the SUPS structure achieves a good relationship between applied external force and the electrical response of the device. Thus, the electrical signal of the SUPS can be modulated by the applied external force. Based on four different structures of triboelectric films, the electrical characterization of the SUPS was measured by applying a vertically compressive force (close to 50 N) with a linear motor on the triboelectric films (Figure 20a). The triboelectric layers integrated by n-Kapton and n-Cu films registered the best electrical output performance of the SUPS. For this case, the maximum output voltage, current, and transferred charge of the device were approximately 109 V, 2.73 mA, and 7.6 nC, respectively. Furthermore, the electrical performance of the device was measured on the radial arteria of a man (24 years old), as shown in Figure 20b. For this second case, the maximum output voltage, current, and transferred charge of the structures of n-Kapton and n-Cu were 1.52 V, 5.4 nA, and 1.08 nC, respectively. Also, the SUPS performance was tested to detect the mechanical oscillations of honeybee wings, which were converted to electrical signals (Figure 20c).

**Figure 21:**
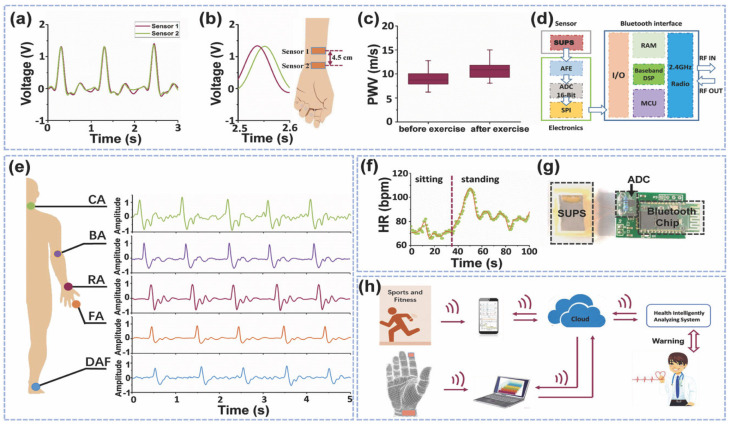
Electrical output variations on different artery regions from the human body and exercise activities, and the elements with big data and cloud of wireless pulse sensor system [111]. (**a**) Output voltage of two SUPS placed on different radial artery regions of a wrist. (**b**,**c**) Output voltage of two SUPS due to radial artery signals before and after an exercise activity. (**d**) Schematic representation of the components in the wireless pulse sensor system. (**e**) Output voltage of SUPS due to signals of different artery regions from a human body. (**f**) Variations of the heart rate during the activity of sitting and standing up. (**g**) Photograph of SUPS, ADC components, and Bluetooth interface of the wireless health monitoring system. (**h**) Schematic representation for online monitoring of cardiovascular signals of patients using the intelligent mobile diagnosis system. Reprinted with permission from [111]. Copyright ©2017, WILEY-VCH Verlag GMbH & Co. KGaA, Weinheim.

Two SUPS devices placed at distinct locations on the radial artery of the wrist of a man (24 years old) were used to measure the pulse wave velocity (PWV), as shown in Figure 21a,b. The PWV variations of this male patient before and after exercise consisting of 500 m jogs were recorded as shown in Figure 21c. Figure 21d depicts a schematic view of the main elements of the wireless pulse sensor system. This system includes the SUPS, analog-digital conversion, storage unit (8M-bit data), and Bluetooth interface. The SUPS output signals can be digitized using the ADC and wirelessly transmitted to a smart phone or laptop with the Bluetooth interface (Figure 21h). Moreover, the SUPS can be adjusted to different human body parts for monitoring the pulse signals of the ankle artery, the finger, the radial artery, the brachial artery, and carotid artery zones, as shown in Figure 21e. Also, the SUPS could detect variations of the heart rates of patients due to different diary movement activities. In the future, this SUPS could be used in self-powered and wearable mobile diagnosis devices for cardiovascular diseases.

**Figure 22:**
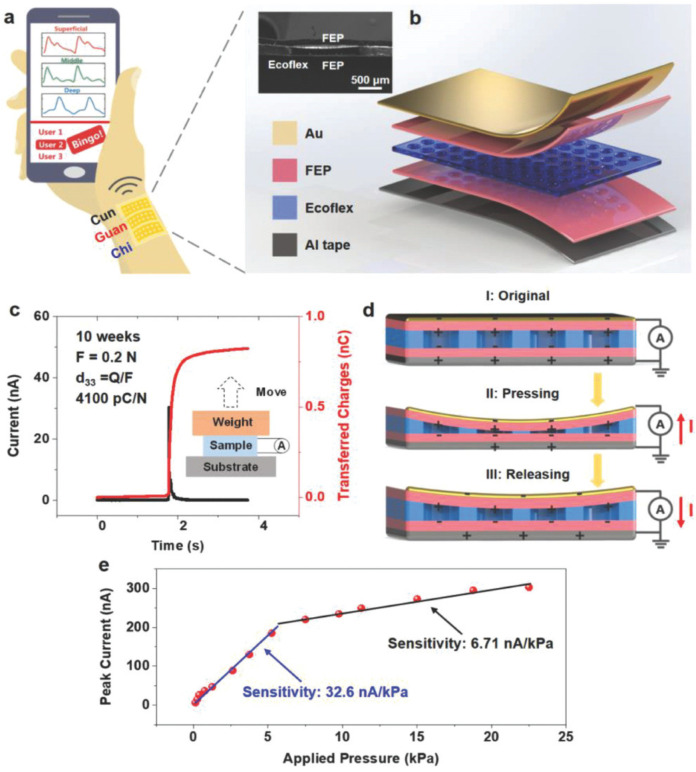
Representation of the sandwich-structure piezoelectret to supply a pulse-sensing system fabricated by Chu et al. [112]. (**a**) Schematic representation of the pulse-sensing system for monitoring pulse signals at the Cun, Guan, and Chi regions of the wrist. These signals could be transmitted to a smart phone. (**b**) Structural configuration of the pulse sensor integrated by the FEP/Ecoflex/FEP piezoelectret layer. (**c**) Experimental results of the equivalent piezoelectric coefficient (d_33_) of the piezoelectret structure of the pulse sensor. (**d**) Working mechanism of the pulse sensor into its stages of pressing (I-II) and releasing (III-I), which induces the flow of electrical currents. (**e**) Experimental results of the sensitivity of peak short-circuit currents generated by a pulse sensor when an external pressure range is applied to it with a frequency of 1.5 Hz. Reprinted with permission from [112]. Copyright ©2018, WILEY-VCH Verlag GMbH & Co. KGaA, Weinheim.

**Figure 23:**
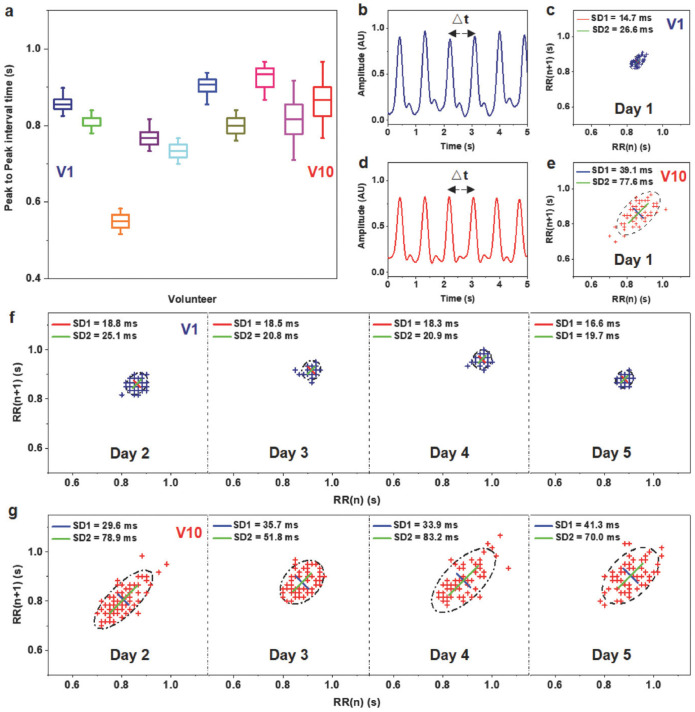
Results of the pulse-sensing system measured from ten volunteers [112]. (**a**) Statistical data of the peak-to-peak pulse wave intervals for the ten volunteers. The ninth and tenth volunteers (V9 and V10) have pulse intervals with larger dispersion compared to the other eight volunteers. It is due to the ninth and tenth volunteers having arrhythmia that was previously diagnosed using ECG tests in hospitals. (**b**) Amplitude of pulse waves and (**c**) corresponding Poincare plots for the first volunteer (V1), who was not diagnosed with arrhythmia. (**d**) Amplitude of pulse waves and (**e**) corresponding Poincare plots for the tenth volunteer, who has arrhythmia. Poincare plots for the (**f**) first and tenth (**g**) volunteers. The volunteer V10 has larger dispersion of pulse wave intervals. Large dispersion of the pulse wave intervals could be related to arrythmia disease. Reprinted with permission from [112]. Copyright ©2018, WILEY-VCH Verlag GMbH & Co. KGaA, Weinheim.

**Figure 24:**
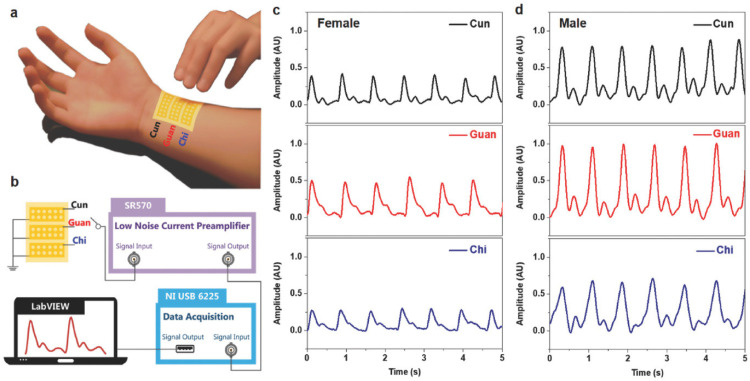
Results of the pulse waves at the Cun, Guan, and Chi regions of the radial artery of the wrist from a human body that were measured employing a three-channel pulse sensing array [112]. (**a**) Representation of the pulse-sensing array placed at the Cun, Guan, and Chi regions. (**b**) Signal processing of the pulse-sensing array. Results of normalized Cun, Guan, and Chi pulse signals for (**c**) a 26-year-old female volunteer, and (**d**) a 28-year-old male volunteer. Reprinted with permission from [112]. Copyright ©2018, WILEY-VCH Verlag GMbH & Co. KGaA, Weinheim.

Chu et al. [112] proposed an active pulse sensing system for monitoring the vibration signals of the human radial artery. This system includes a sandwich-structure piezoelectret with high equivalent piezoelectricity. This structure is composed of fluorinated ethylene propylene (FEP)/Ecoflex/FEP layers. For the initial stage of this structure, the two electrodes (Al and Cu layers) have the same electrical potential. When the structure is deformed and compressed by external pressure, its dipole moment suffers a variation that generates a positive current in the external circuit. The reported system with wireless transmission and big data analyses could operate as a wearable m-Health system (Figure 22a). Figure 23 depicts the results of pulse wave intervals and corresponding Poincare plots measured from ten volunteers. In addition, Chu et al. [112] developed a three-channel pulse sensor array to measure the pulse waves at the Cun, Guan, and Chi pulse regions on the radial artery of the wrist considering the traditional Chinese medicine (TCM) technique (Figure 24). Thus, a pulse-sensing system could detect and collect a large amount of the pulse signals in these three regions for potential applications of big data analyses and diagnoses. The high precision and stability of the proposed system are suitable for potential application as medical assessments, considering the identification of common heart diseases and measurement of blood pressure.

**Figure 25:**
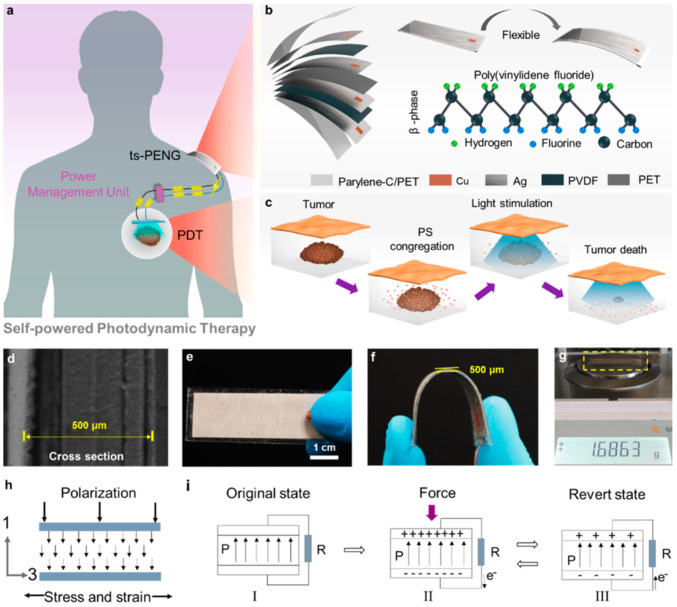
Representation of a self-powered photodynamic therapy system proposed by Liu et al. [113]. (**a**) This system includes a ts-PENG, a PMU, an m-LED, and a photosensitizer. (**b**) Materials of the different layers of the ts-PENG considering the packaging and substrate. (**c**) Representation of the s-PDT used in the apoptotic process of subcutaneous tumor tissue. (**d**) SEM image of the cross section of the st-PENG. (**e**) Image of the initial state of the ts-PENG. (**f**) Image of the bending state of the ts-PENG. (**g**). Weight of the ts-PENG. (**h**) Schematic view of the d_31_ operating mode of the PVDF film. (**i**) Operating principle of the PVDF film. Reprinted with permission from [113]. Copyright ©2020, American Chemical Society.

Liu et al. [113] designed a self-powered photodynamic therapy (s-PDT) system for potential application in cancer treatment. This s-PDT system is composed of a wearable twinning structure piezoelectric nanogenerator (ts-PENG), a power management unit (PMU), a miniaturized LED (m-LED), and a photosensitizer (PS) (Figure 25). This nanogenerator converts biomechanical energy into electrical energy to power a m-LED. This m-LED is used to stimulate light over tumor tissues, which inhibits tumor growth. The performance of this LED is controlled by the PMU with dimensions of 1.7 cm × 4.8 cm × 1.3 cm. This PMU can drive two irradiation modes: intermittent continuous light stimulation (ICLS) and pulsed light stimulation (PLS). For the ICLS mode, the tumor tissue can suffer strong radiation intermittently, which renders it dead. For the PLS case, small residual tumors can be destroyed by applying continuous low-dose irradiation.

**Figure 26:**
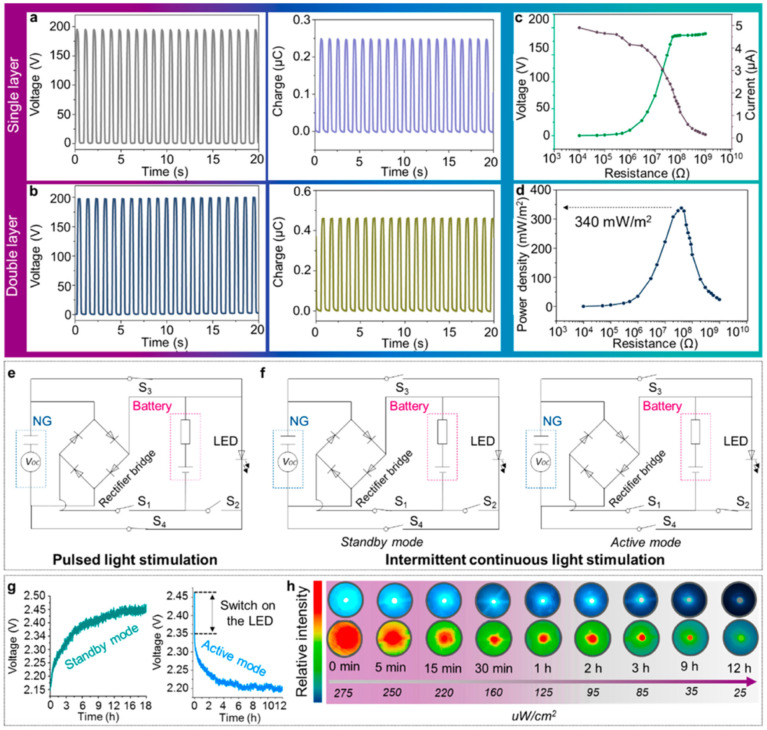
Performance of the ts-PENG designed by Liu et al. [113]. The open-circuit voltage and short-circuit transferred charge of the PENG with (**a**) single and (**b**) double PVDF film. (**c**) Voltage and current and (**d**) power density of the ts-PENG under different load resistances between 10 kΩ and 10 GΩ. (**e**,**f**) Schematic representation of the irradiation modes of PLS and ICLS. (**g**) Charging and discharging voltage of the button cell by the ts-PENG and LED, respectively. (**h**). Results of the light intensity of LED as a function of time. Reprinted with permission from [113]. Copyright ©2020, American Chemical Society.

**Figure 27:**
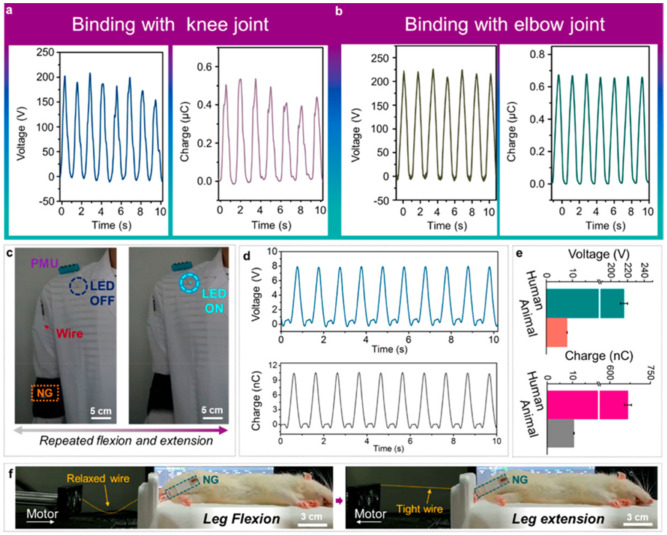
Experimental results of the output performance of the ts-PENG tested in human and rat models [113]. The open-circuit voltage and short-circuit transferred charge of the ts-PENG placed on (**a**) the knee joint and (**b**) elbow joint. (**c**) Test of the ts-PENG system to light an LED. (**d**) The open-circuit voltage and short-circuit transferred charge of the ts-PENG attached to the leg of a rat model. (**e**) Average results of the open-circuit voltage and short-circuit transferred charge of the ts-PENG tested on the human and rat models. (**f**). Image of the ts-PENG tested on the leg of the rat model. Reprinted with permission from [113]. Copyright ©2020, American Chemical Society.

**Figure 28:**
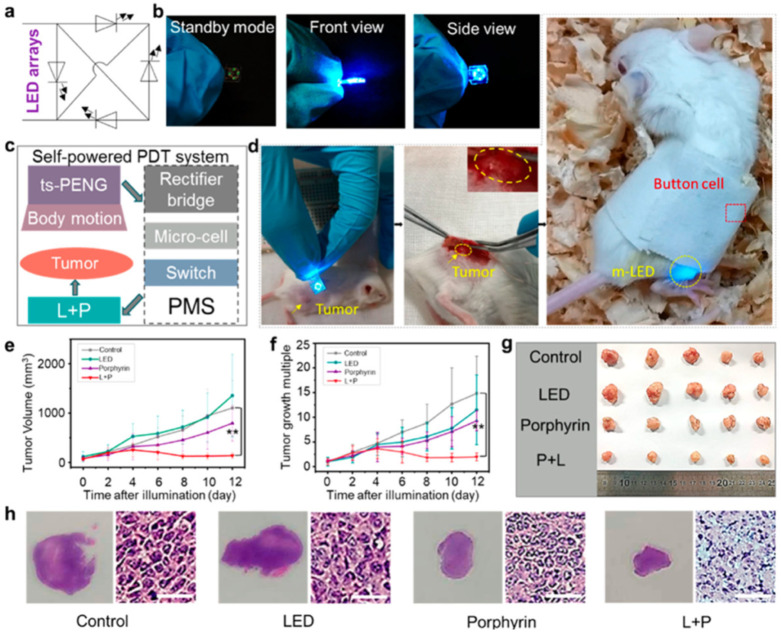
Experimental results of intermittent continuous light stimulation applied to a tumor in vivo [113]. (**a**) Electrical diagram of LED array. (**b**) Different views and states of the LED array packaging by PDMS. (**c**) Schematic diagram of the self-powered PDT system. (**d**) Application of the self-powered PDT system to inhibit a tumor model on a rat. (**e**) Variation of the tumor volume using a control group without any treatment and experimental groups cultured with porphyrin, LED, and LED + porphyrin. (**f**). Results of tumor growth multiplication during 12 days of a control group without any treatment and experimental groups cultured with porphyrin, LED, and LED + porphyrin. (**g**,**h**) Photographs of the tumors and pathological section (50 μm of scale bar) obtained from different experimental groups. These results were measured after 12 days. Reprinted with permission from [113]. Copyright ©2020, American Chemical Society.

The ts-PENG structure developed by Liu et al. [113] is formed by a Parylene-C/PET packaging layer, a PET substrate, and a double piezoelectric layer of PVDF and Ag electrodes. The ts-PENG achieves the open-circuit voltage (V_oc_) and short-circuit transferred charge (Q_sc_) close to 200 V and 0.46 μC (Figure 26b). Furthermore, the ts-PENG reaches 340 mW/m^2^ under load resistances between 10 kΩ and 10 GΩ (Figure 26d). Moreover, the wearable ts-PENG was tested on human and rat models (Figure 27). First, the ts-PENG was attached on the knee joint and the elbow joint of a human body. The maximum values of the V_oc_ and Q_sc_ of the ts-PENG placed on knee joint and the elbow joint achieve 200 V, 220 V, 0.5 μC, and 0.65 μC, respectively. Also, an LED can be lighted using the biomechanical energy harvested by the wearable ts-PENG. In future applications, the PMU of the ts-PENG could drive the LED to apply both PLS and ICLS irradiation modes to tumors of the human body. On the other hand, the ts-PENG was positioned on the leg of a rat to convert its motion into electrical energy. A linear motor was used to pull the leg and simulate its mechanical motion. For this rat model, the maximum results of the V_oc_ and Q_sc_ of the ts-PENG are close to 8 V and 10.5 nC (Figure 27d). On the other hand, the s-PDT system was used for inhibiting tumors in vivo with intermittent continuous light stimulation (Figure 28). Finally, the ts-PENG offers advantages such as simple structural configuration, high flexibility, good stability, light weight, and long service life. These ts-PENG characteristics are suitable for self-powered wearable medical devices.

## 4. Challenges and Perspectives

This section describes various challenges and perspectives of piezoelectric and triboelectric nanogenerators to power future healthcare monitoring devices, including the topics of design, materials and fabrication processes, signal processing, output performance, and reliability.

### 4.1. Design

The nanogenerator design process is key to developing better piezoelectric and triboelectric structural configurations that optimize energy harvesting to power healthcare monitoring devices. For each potential medical device, the design of a nanogenerator must examine the main performance requirements and limitations of this device, optimal materials and manufacturing process, surrounding conditions (e.g., temperature, relative humidity, pressure, dust, and so on), encapsulated type, signal processing components, and the characteristics of the original energy sources (e.g., biomechanical, wind, mechanical vibrations, and so on). For instance, nanogenerators can harvest biomechanical motion to power medical devices. However, optimal energy harvesting from human body motion has great challenges. In order to develop efficient and stable nanogenerators attached to the human body, the design of nanogenerators must consider scalable, wearable, durable, and stretchable materials. In the coming years, smart textile-based nanogenerators could be incorporated into clothing using piezoelectric or triboelectric materials with special characteristics such as being wearable, stretchable, durable, and washable. These nanogenerator types could power smart sensors that monitor real-time signals on the health status of patients. By using the IoMT, these signals could be transmitted to medical hospitals or doctors. In addition, future nanogenerators could be attached to different parts of the human body, which will require designs of electromechanical components composed of materials with high flexibility, high reproducibility, easy fabrication, and friendly performance. Future implantable self-powered medical devices will require nanogenerators with biocompatible packaging materials.

To achieve optimal designs for the output performance of nanogenerators, multi-optimization techniques can be implemented, including the various objective functions and constraints. For instance, the output power density and cost of a nanogenerator can be the objective functions to be maximized and minimized. The constraints equations of a nanogenerator can consider its size, service time, reliability parameters, and so on. For these optimization models, the mechanical and physical properties of the materials of the nanogenerator must be known.

### 4.2. Materials and Fabrication Processes

The output performance and reliability of piezoelectric and triboelectric nanogenerators for monitoring healthcare devices depend on the suitable selection of their materials and manufacturing processes. These materials should have electromechanical properties that allow for the development of nanogenerators with the benefits of cost-effectiveness, wearability, flexibility, easy fabrication, stability, and robust performance. Furthermore, the optimal selection of materials can be useful to fabricate smart nanogenerators with both functions for energy scavenging and working as active/self-powered sensors, which reduce their size and weight. Moreover, smart nanogenerators can be eco-friendly to the environment, eliminating the use of harmful materials from conventional batteries.

Recent investigations of nanogenerators have taken into account organic or waste materials, including chitin, spider silk, tomato, rice paper, peanut, walnut, pistachio, almond, fish gelatin, fish bladder, eggshell, sunflower husks, garbage soda cans, silk fibroin, and so on [114,115,116,117,118,119,120,121]. For these materials, two research challenges are the analytical modeling and experimental measurement of their piezoelectric and triboelectric transduction mechanisms. In addition, future investigations must consider standard rules and tests to measure the performance and reliability of nanogenerators. Another challenge is the development of scalable manufacturing processes for nanogenerators, which decrease costs and satisfy the future commercial market.

### 4.3. Signal Processing

Generally, the output voltage and current of triboelectric nanogenerators are not direct current (DC) signals. Due to this limitation, triboelectric nanogenerators need rectifier circuits to convert their AC output signals into DC signals. This DC voltage can be stored using capacitors or batteries to power the monitoring healthcare devices. A research challenge in this topic is the development of cost-effective energy storage systems coupled with nanogenerators.

Recent investigations [122,123,124] on nanogenerators have included novel electrical interfaces of high efficiency and minimum power consumption. The electrical interfaces could be self-powered and include cold-start circuit architectures [125,126]. In addition, the electrical interfaces could incorporate a small footprint to decrease the size of the nanogenerators. For this, the Application-Specific Integrated Circuit (ASIC) could be implemented.

### 4.4. Reliability

**Figure 29:**
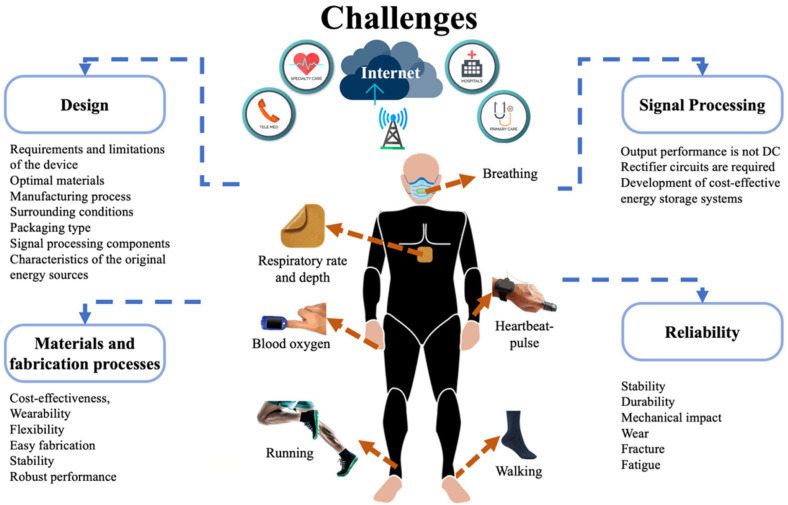
Summary of challenges of piezoelectric and triboelectric nanogenerators in IoMT electronic devices.

The implementation of reliability standard tests and rules is required to measure the safe and stable performance of nanogenerators for medical applications. The reliability analyses of the electromechanical performance of nanogenerators under different environments and operating conditions are required to study their stability, durability, and working capability. These reliability tests can provide the main electromechanical failures of the nanogenerators due to various factors, such as high relative humidity, large temperature variations, dust, mechanical impact, wear, crack growth, fatigue, and so on. Furthermore, the different packaging types and signal-processing components of nanogenerators must be included in the reliability tests. In order to improve the reliability of nanogenerators, the designers could consider simple operating mechanisms, minimum electrical and structural components, robust and durable materials, safe packaging, and so on. Figure 29 depicts various challenges of the piezoelectric and triboelectric nanogenerators for their application in IoMT electronic devices.

## 5. Conclusions

Recent progress on piezoelectric and triboelectric nanogenerators for self-powered monitoring healthcare devices was reviewed. The descriptions of the working principle, materials, and fabrication processes of a variety of these nanogenerators were reported. Discussions on the output electrical performance of piezoelectric and triboelectric nanogenerators are included. Furthermore, we presented the signal processing components and packaging types of different nanogenerators. The design, materials, and electromechanical behavior of different piezoelectric and triboelectric nanogenerators for biomechanical energy harvesting to power potential medical applications were considered. Also, we proposed some challenges and perspectives of these nanogenerator types, including the design, materials and manufacturing processes, signal processing, and reliability.

## Figures and Tables

**Table 1:** Comparison of advantages and disadvantages of different devices used in the energy harvesting and storage process.

Device Type	Advantages	Disadvantages	Ref.
Metal-air batteries	High energy density, low cost, flat discharge voltage, and high safety	Performance is affected by environmental conditions, dendrite formation on the anode, carbonation of alkaline electrolyte, and limited range of operating temperature	[30,32]
Supercapacitor	High energy density, high specific surface area, long cycle life, and good conductivity and stability	Capacitance and charge storage depend on the employed electrode materials and non-simple fabrication process	[33,34,35]
Piezoelectric nanogenerator	Simple structure and easy fabrication process, good electromechanical stability, and non-complex signal processing system	Performance depends on the properties and structural configuration of the piezoelectric material	[36]
Triboelectric nanogenerator	High electrical performance, compact structure, simple working principle, low-cost materials, and good electrical stability	Wear of triboelectric material by friction, and performance depends on the properties and working mode of the triboelectric film	[36]

## Data Availability

Not applicable.
